# Running the Gauntlet: Regional Movement Patterns of *Manta alfredi* through a Complex of Parks and Fisheries

**DOI:** 10.1371/journal.pone.0110071

**Published:** 2014-10-22

**Authors:** Elitza S. Germanov, Andrea D. Marshall

**Affiliations:** Marine Megafauna Foundation, Truckee, California, United States of America; Virginia Commonwealth Univ, United States of America

## Abstract

Manta rays (Genus *Manta*) are economically important for fisheries and tourism in Indonesia. These species have been listed by the International Union for the Conservation of Nature Red List as Vulnerable to extinction; therefore, human exploitation of manta rays must be regulated. A better understanding of the habitat use and movement patterns of manta rays in Indonesia is needed in order to employ effective conservation measures. To gain better insight into the movements of *Manta alfredi* we used ‘Manta Matcher’, an online database with an integrated automated matching algorithm, to compare photographs from 2,604 encounters of *M. alfredi* collected by recreational divers and dive operators throughout Indonesia over a nine-year period. This photographic comparison revealed that manta rays migrated between regional sanctuaries such as Nusa Penida, the Gili Islands, and the Komodo National Park (up to 450 km straight-line distance). The areas between these sanctuaries are heavily fished and trafficked by ships, and when manta rays travel through these regions they risk being fished and injured by ship strikes. These long-range manta ray movements suggest connectivity between *M. alfredi* populations in neighboring islands and raise concerns about the future management of regional populations. It is recommended that a national conservation strategy be developed to protect the remaining populations in the country.

## Introduction

Manta rays (genus *Manta*) are large pelagic filter-feeding, elasmobranchs that are economically important for both tourism [1] and fisheries throughout many parts of their range [2], [3]. Manta rays are classified as Vulnerable to extinction according to the International Union for Conservation of Nature (IUCN) Red List [4], [5] due to their slow growth rates, low fecundity [6] and globally rising pressure from fisheries [3], [7]. Manta rays aggregate at many locations within the Indonesian archipelago [3], [7], [8]. Indonesia has the fourth highest number of known tourism sites for viewing manta rays worldwide and rates as one of the top countries in the world for manta ray watching tourism [1]. Manta ray tourism in Indonesia is valued at over $15 million USD per year [1]. Indonesia also has some of the most aggressive targeted fisheries for manta rays [2], [3], [9], [10]. Fisheries most negatively impact the sizes of manta ray populations [1], [3]. The reduced number of manta rays will likely negatively impact the burgeoning manta ray tourism industry [1].

To protect the manta ray tourism economy in Indonesia, three manta ray sanctuaries were established in Raja Ampat (11,655 km^2^), West Manggarai including Komodo National Park (7,000 km^2^), and Nusa Penida (200 km^2^). In 2013, manta rays were listed on Appendix II of the Convention on International Trade in Endangered Species (CITES) as a preemptive measure against potentially unsustainable fisheries. As a member country, party to this convention, in early 2014 Indonesia further declared manta rays a protected species, prohibiting the catch of *Manta* species throughout the entire exclusive economic zone of the country (Seas and Fisheries decree number 4). Indonesian and CITES regulations will help curb fishing related threats within the region, but sound conservation strategies are also needed to protect decreasing manta ray populations [7]. Information about the distribution, movement patterns, and population sizes of manta rays is needed in order to create effective conservation management strategies.

**Figure 1:**
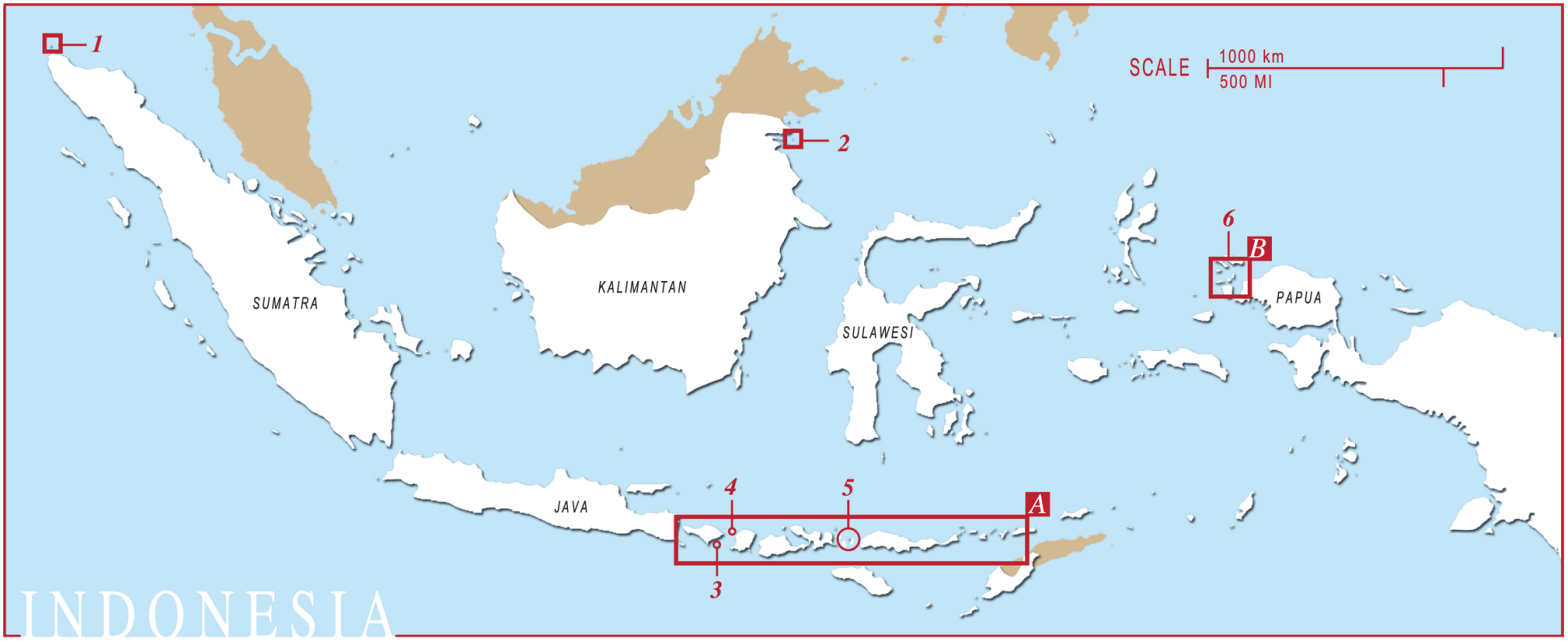
The locations of *M. alfredi* encounters in Indonesia submitted to Manta Matcher. Sites are numbered as follows: 1) Pulau Weh 2) Palau Sangalaki 3) Nusa Penida 4) Gili Islands 5) West Manggarai & Komodo National Park 6) Raja Ampat. (A) Connectivity area and commercial fisheries area (see Figure 2 for enlargement). (B) Region surveyed in Raja Ampat (see Figure 3 for enlargement).

Understanding the migratory range of manta rays (*M. alfredi*) in Indonesia is particularly important, as major fisheries often occur near to marine sanctuaries. A previous study using acoustic telemetry, showed that *M. alfredi* (referred to then as *M. birostris*) exhibit considerable site fidelity through localized re-sightings within areas of the Komodo National Park [11]. This study also demonstrated that there was considerable movement (of up to approximately 33.5 km apart) between the individual sites that coincided with seasonal monsoon-shifts [11]. While this was a good preliminary effort to learn more about the habitat use of *M. alfredi* within the Komodo National Park, this study was spatially limited by the technology used leaving many unanswered questions. For instance, (34%) of the tagged individuals spent a minimum of 2 months and a maximum of 9 months outside of the range of the acoustic receiver array (which was set up within the park) suggesting that *M. alfredi* may also travel to and spend significant amounts of time in other areas [11]. Recent studies have reported *M. alfredi* movements greater than 380 km (straight-line distance) in places like Mozambique and Australia [5], [8], [12]–[14]. Major fishing grounds and known manta ray landing ports, Tanjung Luar, Lombok [3] and Lamakera [3], [10], are within 380 km of *M. alfredi* sanctuaries in West Manggarai and Nusa Penida (Figure 1). Thus it is plausible that the migratory range of *M. alfredi* may overlap with heavily fished areas in Indonesia.

The aim of this study was to determine the connectivity and rate of movement of manta rays between the manta ray sanctuaries of Nusa Penida, West Mangarrai province of Flores including the Komodo National Park and Raja Ampat. This study used a large-scale photo-identification (photo-ID) approach to monitor manta ray movements using publically contributed photographs. Photo-ID methodology, where by animals are identified using their unique ventral patterning, has been successfully used in a large number of manta ray population and movement studies to date [6], [8], [13], [15]–[17]. Photo-ID data usage does have limitations [18], but it is still a cost-effective and minimally invasive tool for gathering large amounts of data, especially when coupled with citizen science efforts [8], [12], [13], [19], [20]. This is the first study to use ‘Manta Matcher’, a global collaborative photo-ID matching database, to compare regional photo-ID databases. ‘Manta Matcher’ uses an automated biometrics algorithm to accelerate the photo-identification process [21]. Using ‘Manta Matcher’ enabled the consolidation of existing citizen-scientist collected data in Indonesia into an easily accessible database, thereby uncovering long-distance *M. alfredi* movements and highlighting the need for more effective conservation strategies of this migratory species.

## Methods

### Study locations

**Figure 2:**
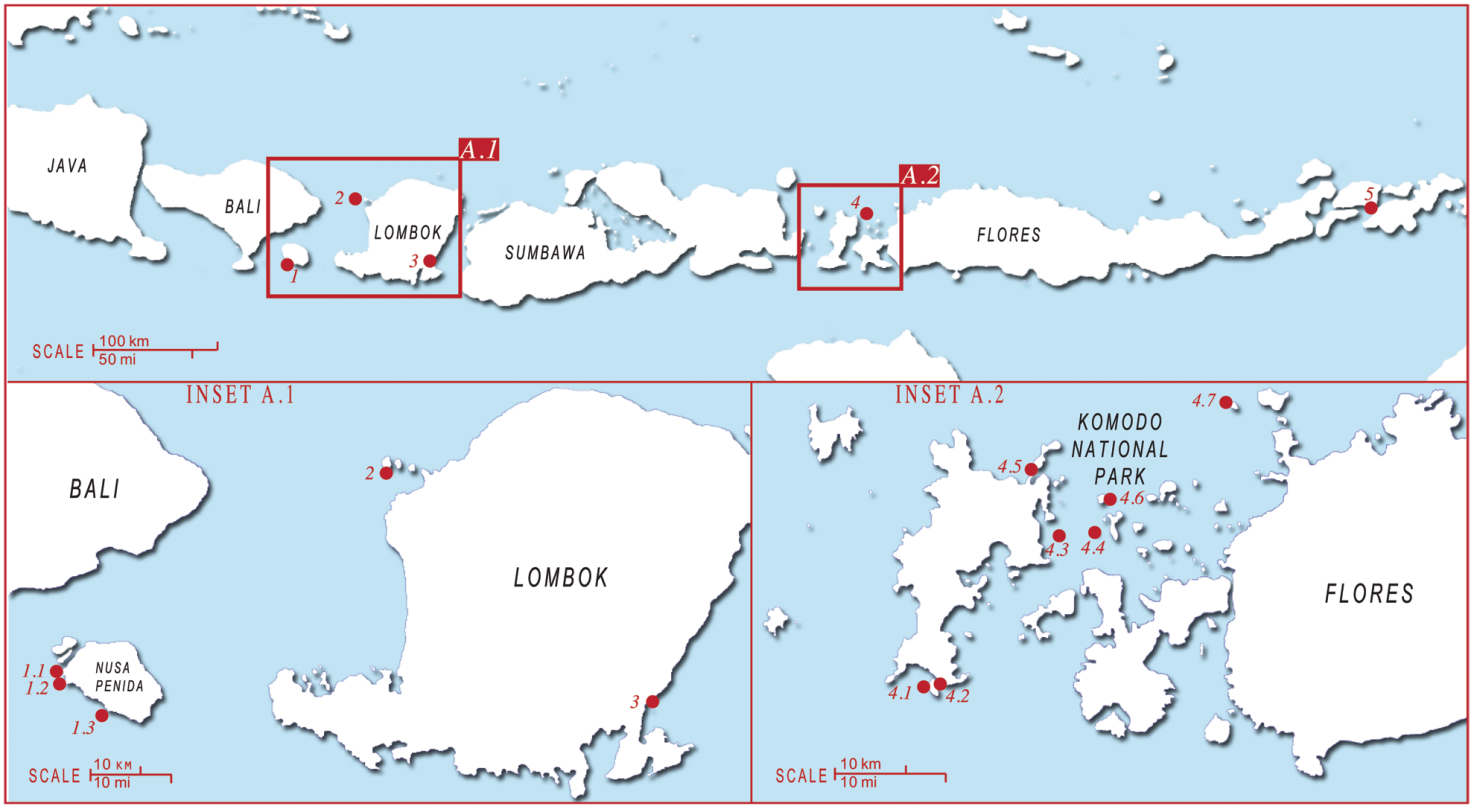
The connectivity area encompassing Nusa Penida, West Manggarai & Komodo regions and the nearby commercial manta ray landing ports. Regions and fishing ports are numbered as follows: 1) Nusa Penida 2) Gili Islands 3) Tunjung Luar fishing port 4) Komodo National Park 5) Lamakera fishing port. (A) Manta ray monitoring sites in Nusa Penida and Lombok and nearby Tujung Luar fishing port: 1.1) Small Manta Bay 1.2) Big Manta Bay 1.3) Manta Point 2) Gili Islands 3) Tanjung Luar fishing port. (B) Manta ray monitoring sites in West Mangarrai and Komodo National Park: 4.1) Manta Alley 4.2) German Flag 4.3) Karang Makassar/Manta Point 4.4) Mawan 4.5) The Cauldron 4.6) Tatawa Besar 4.7) Sabolon Kecil.

**Figure 3:**
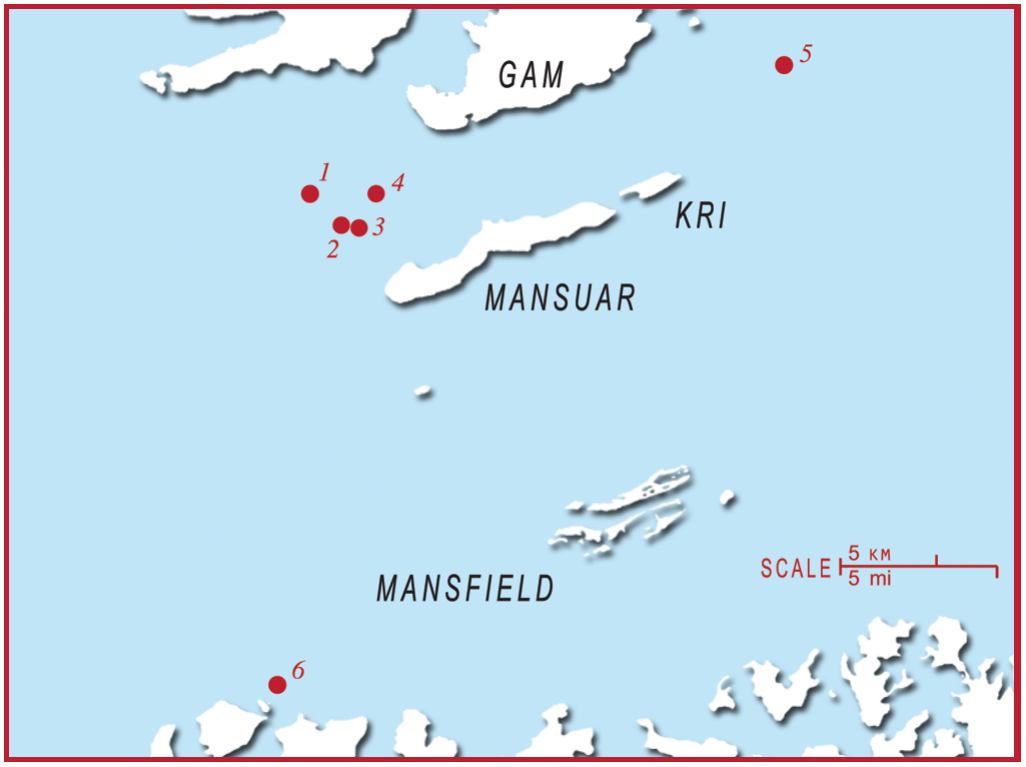
A map of the Raja Ampat region to indicate the location of different monitoring sites. Sites are numbered as follows: 1) Manta Sandy 2) Jetty Arborek 3) Blue Magic 4) Manta Ridge 5) Manta Heaven 6) Dayang.

Photographs of the ventral surfaces of individual *M. alfredi* were collected year round by local dive operators and recreational divers at various manta ray aggregation sites throughout Indonesia (Figures 1, 2 and 3) from 2006–February 2014. Data that recently became available on manta ray INNLP0229A in both June and July 2014 were also included in this study. Photographs were collected opportunistically with effort mostly concentrated at sites in Nusa Penida (NP), West Mangarrai & Komodo National Park (WM & K) and Raja Ampat (RA) regions (Table 1). Limited data from Pulau Weh in Aceh province, Pulau Sangalaki in Kalimantan and the Gili Islands was also collected. Monitored sites represent a range of habitat used by *M. alfredi,* including cleaning stations, feeding aggregation areas, and reproductive grounds.

**Table 1:** The survey effort in Nusa Penida, West Manggarai & Komodo, and Raja Ampat regions including sighting records and number of individuals identified.

Region	Year	NusaPenida	West Manggarai& Komodo	RajaAmpat	Total
Total SightingRecords	2006–14	2007	426	171	2604
Yearly SightingRecords	2006–10	83	-	-	83
	2011	84	49	-	133
	2012	544	37	-	581
	2013	1092	329	53	1474
	2014	203	11	113	327
*M. alfredi*identified (N)	2006–14	417	303	100	820

The majority of photographic data comes from the monitoring of 16 separate sites within the NP, WM & K and RA regions. Photographs within the NP region were mostly collected from three nearby sites: Small Manta Bay, Big Manta Bay and Manta Point (Figure 2 inset A.1). The two furthest sites are located at approximately 12.4 km straight-line distance apart. The photographs submitted for the WM & K region were collected from 7 sites out of 15 regularly visited locations (Figure 2 inset A.2). The majority of photographs available for the WM & K region were collected from Karang Makassar and Mawan Island, both located in the central-north region of the Komodo National Park, and Manta Alley to the south of Komodo Island. Mawan Island and Manta Alley are respectively located 4.4 km to the east and approximately 33.5 km (straight line) to the south of Karang Makassar (Figure 2 inset A.2). Most of the monitored sites in the RA region are in close proximity to one another (Figure 3). Manta Sandy, Manta Ridge, Arborek, and Manta Heaven, are all within a 3.5 km straight-line distance. Two further sites from which photographs were obtained, Blue Magic and Dayang, are respectively located approximately 23 km south and 24 km east from Manta Sandy.

### Photographic identification

Manta rays can be individually distinguished by their unique ventral markings [15], [16], allowing for the application of photo-ID techniques. Photographs of the standardized area [2], [8], [13], [15]–[17], [22] on the ventral surface of manta rays were taken from still photography with or without flash, or from video screen grabs, as described in [8], [13], [15], [16]. Species identification was performed using key morphological features described by Marshall *et al.* (2009) [22]. Whenever possible, manta rays were sexed and their maturity status assessed based on clasper size and/or clasper scarring for males, and pregnancy bulge and/or presence of pectoral fin mating scars for females as outlined in [6]. Other distinguishing features such as color morphology, scars and injuries were also noted.

### ‘Manta Matcher’

Photographs of individual manta ray encounters were submitted online by the authors and volunteers to ‘Manta Matcher’ (www.mantamatcher.org), the Wildbook for manta rays created by WildMe. Individual encounter profiles were completed for each entry (see Table S1 for encounter profiles of key individuals), which included the photographer and/or submitter, date, location and GPS coordinates of sightings. Whenever possible sex, maturity status, color morphology and any other distinguishing characteristics were added. Only clear, good-quality images in which individual rays could be identified using the standardized ventral spot pattern area were submitted to the ‘Manta Matcher’ biometrics algorithm [21]. When necessary images were first post processed using Adobe Lightroom 3.3 or Photoshop CS5 to enhance visualization of the ventral spot patterns by adjusting brightness and contrast prior to uploading. Images were cropped to isolate the critical identification region (the gill area and abdomen) in a head up orientation [21]. Newly submitted photographs were compared against both the global and regional Indonesian database of submitted images. Overall confidence of results in a scale of 1 (good) and 0 (poor) were automatically generated for every query along with a ranked list of matched hits [21]. Manta rays were subsequently matched by eye from the generated list of ranked hits allowing for regional and global comparisons. Screen grabs of positive matches were saved electronically.

### Ethics statement

The authors deemed that the nature of the work (analysis of publically contributed photographs taken opportunistically by recreational divers) did not require any approval or permits regarding human or animal ethics. When photographs came from areas within marine sanctuaries or national parks the burden of possession of suitable diving and entrance permits was placed on the individual submitting the photographs.

## Results

### Identified Individuals

A total of 820 ­individual *M. alfredi* were identified from 2,604 sightings within the NP, WM & K and RA regions of Indonesia between 2006 and February 2014 (Figure 1). Re-sighting data on one individual (INNLP0229A) sighted in June and July 2014 were also included. Specific sighting rates are listed in Table 1. In the WM & K region 100 out of 303 identified manta rays (33.1%) were sighted more than once, an average of 2.7 times per individual during the study period. In the NP region 274 out of 419 manta rays (65.4%) identified during the study period were sighted more than once, an average of 6.9 times per individual. During the study period 100 individual manta rays were positively identified in the RA region. In this region 30 out of 100 manta rays (30%) were sighted more than once, an average of 3.1 times per individual.

### Long-range Movements and Regional Connectivity

**Figure 4:**
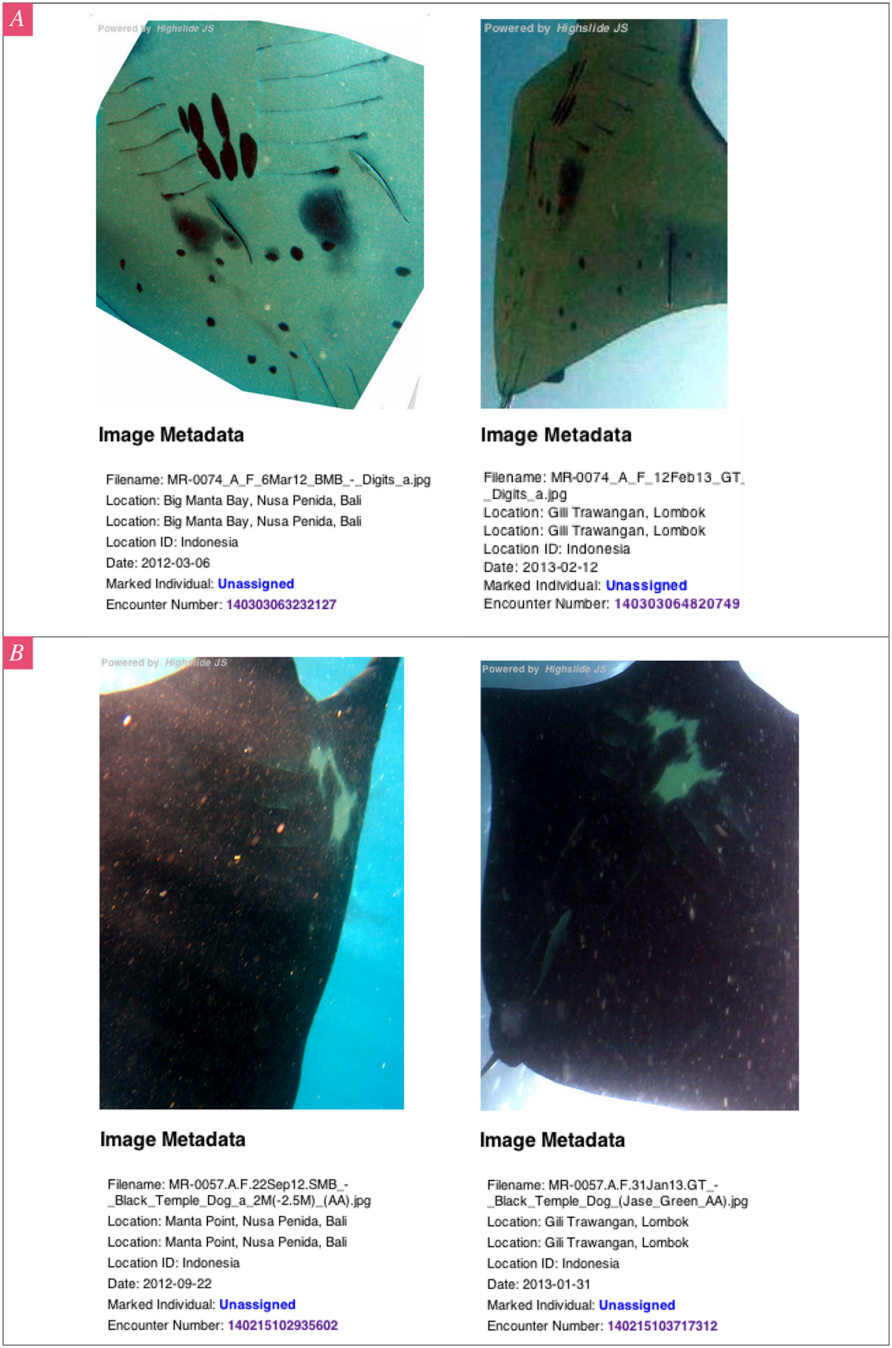
Screen grabs of photographs uploaded to the ‘Manta Matcher’ online database documenting *M. alfredi* movements from Nusa Penida (NP), Bali to Gili Islands (GI), Lombok. A. Manta ID code INNLP0074A. B. Manta ID code INNLP0057A.

The photo-ID comparisons revealed long-range and inter-regional movements of individual manta rays. In the RA region, one male manta ray (INRA0050A) moved between Manta Sandy and Dayang within 3 days and returned to Aborek within 5 days later, a roundtrip straight-line distance of approximately 48 km within 8 days (Figure 3). Furthermore, two female manta rays (Figure 4A, B and Table 2) sighted in the NP region were later sighted in the waters off the Gili Islands, Lombok (Figure 2 inset A. 1), a minimum straight-line distance of approximately 80 km within 131 days (INNLP0057A) and 343 days (INNLP0074A). One of these individuals (INNLP0074A) also subsequently made a return journey back to NP within 38 days.

**Table 2:** The sighting records of individual rays migrating between Nusa Penida and Gili Islands or West Manggarai & Komodo regions.

Identification #	NusaPenida	GiliIslands	WestManggarai& Komodo	Time betweenResightings(days)	ApproximateDistance(km)
INNLP0031A	2009-Aug-18⇒		⇒2013-Apr-03	1324	450
	2013-Sep-07⇐		⇐2013-Apr-03	157	450
INNLP0057A	2012-Sep-22⇒	⇒2013-Jan-31		131	80
INNLP0059A	2008-Dec-25⇒		⇒2013-Mar-24	1550	450
	2013-Jun-03⇐		⇐2013-Mar-24	72	450
	2013-Dec-07⇒		⇒2014-Jan-09	33	450
INNLP0074A	2012-Mar-06⇒	⇒2013-Feb-12		343	80
	2013-Mar-22⇐	*2013-Feb-12		38	80
INNLP0229A	2012-Sep-13⇒		⇒2013-Jun-10	270	435
	2014-Jul-18⇐		⇐2014-Jun-04	44	435

Arrows highlight direction of movements.

⇒ west to east movements;

⇐ east to west movements.

**Figure 5:**
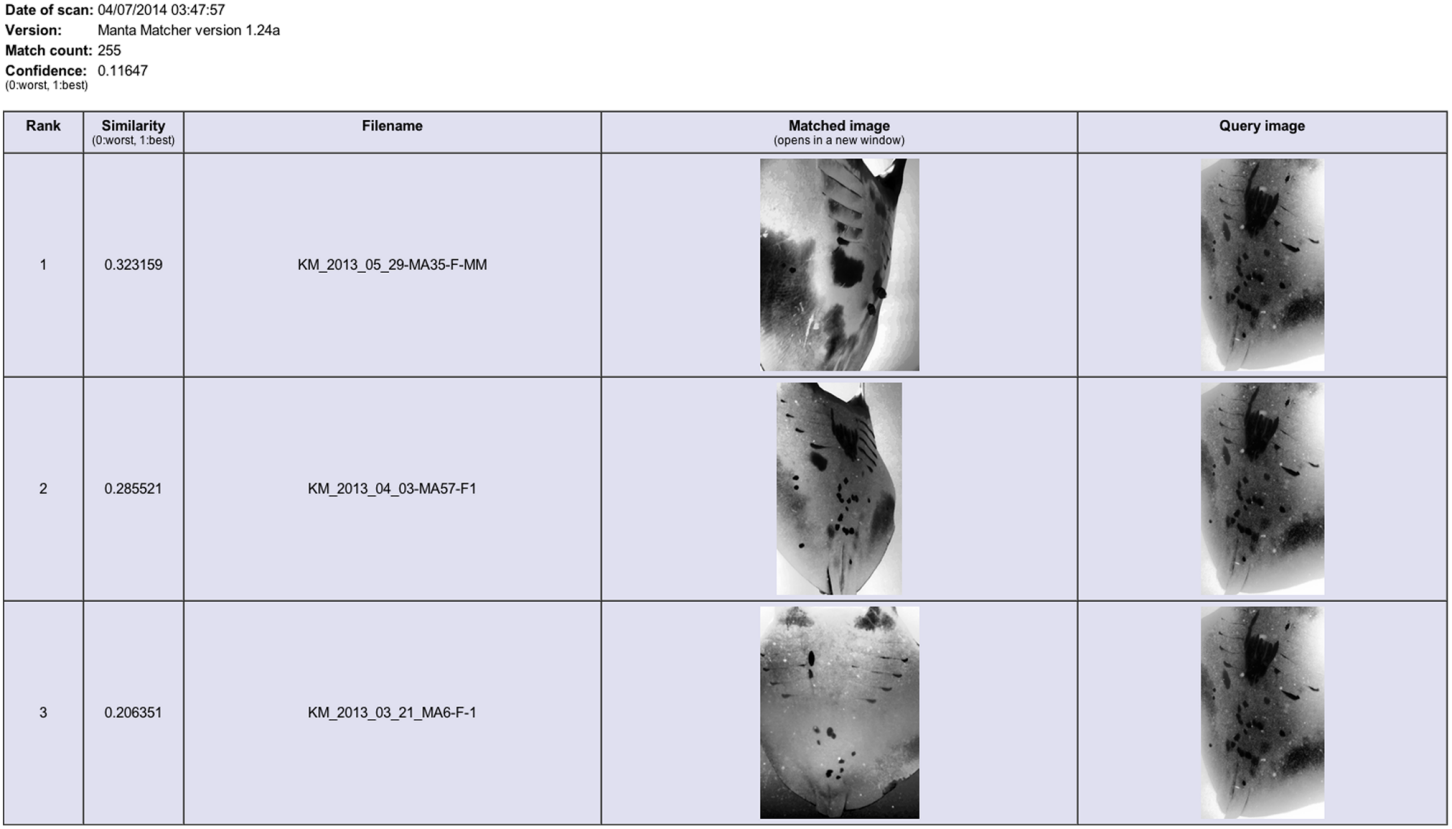
The ‘Manta Matcher’ matching algorithm screen grabs for INNLP0031A showing the top three matched images. Ranked hit 2 (re-sighting in WM & K) is a true match for the query image of ray INNLP0031A (initial sighting in NP).

**Figure 6:**
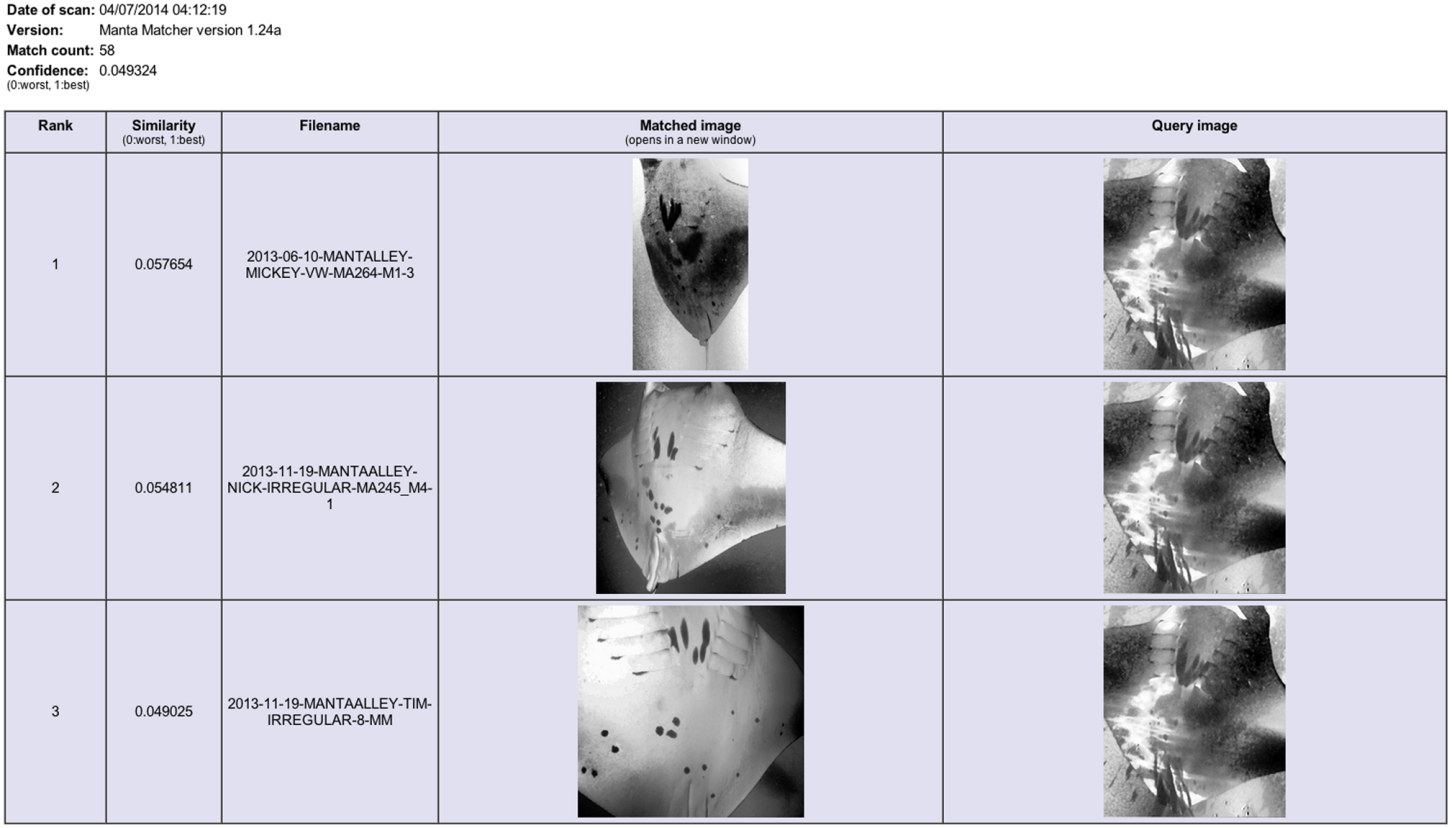
The ‘Manta Matcher’ matching algorithm screen grabs for INNLP0229A showing the top three matched images. Ranked hit 1 (re-sighting in WM & K) is a true match for the query image of ray INNLP0229A (initial sighting in NP).

**Figure 7:**
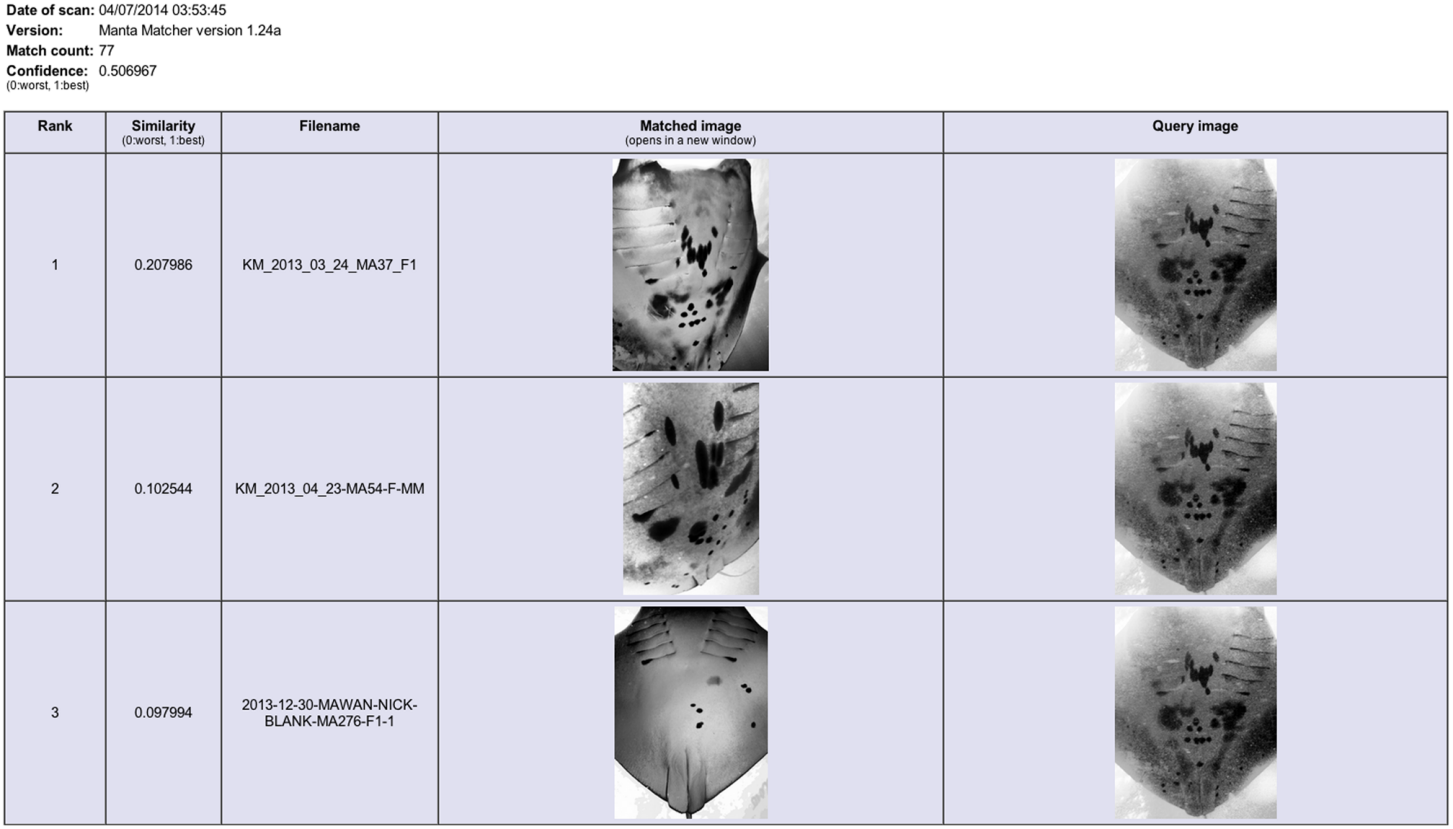
The ‘Manta Matcher’ matching algorithm screen grabs for INNLP0037A showing the top three matched images. Ranked hit 1 (re-sightings in WM & K) is a true match for the query image of ray INNLP0037A (initial sighting in NP).

Moreover, during the study period, three manta rays (Figures 5, 6 and 7), over seven occasions (Table 2), moved between NP and WM & K, a straight-line distance of approximately 450 km (Figure 2). One of these manta rays, a mature female (INNLP0031A), moved from NP to WM & K and then back again to NP (Figure 5), the fastest of the migrations occurring in 157 days or less (Table 2). This manta ray was then re-sighted in the NP region on several occasions during the remainder of the study period but was not re-sighted back in the WM & K region. A mature male ray (INNLP0229A) made this inter-regional journey from NP to the south region of WM & K and then back to NP with the fastest of the migrations occurring in 44 days or less (Figure 6 and Table 2). The fastest migration on record was a mature female ray (INNLP0059A), which traveled from NP to WM & K twice during the study period (Figure 7), once within a 33-day period (Table 2).

## Discussion

### Movement Patterns of *M. alfredi* in Indonesia

A collaborative regional photo-ID database comparison indicated that certain individual manta rays might regularly move between aggregation sites within the manta ray sanctuaries of WM & K, NP and Gili Islands. These results support studies in other parts of the world [8], [11], [13], [16], [17] that have indicated the potential for regional mobility in this species. Moreover, data from this study support the growing concept that *M. alfredi* are capable of rapid, long distance migrations. While to date no international movements have been recorded, the round-trip migrations exhibited by manta rays in southern-central Indonesia suggest that at least certain individuals would be capable of international exchange in some parts of their range. Considering their global conservation status and CITES listing it may be appropriate to include *M. alfredi* on the Convention for Migratory Species Act (CMS) to help support regional management plans between neighboring countries.

Long-term studies in Australia [8], [12], [13], Hawaii [17], Maldives [16] and Mozambique [15], have all noted highly localized re-sighting rates for *M. alfredi*, clear diurnal patterns in their inshore and offshore habitat use, and in some cases seasonal or cyclical migratory behavior. The present study showed that some manta rays are making inter-island movements of up to 450 km straight-line distance within the Indonesian Archipelago being one of the furthest reported straight-line distance migrations for this species. In one specific case a movement of this distance was made in as little as 33 days, the fastest straight-line movement for this species on record. *Manta alfredi* have been documented to make long-distance movements, with the longest traveled straight-line distance reported using photo-ID to be 650 km over a six-month period [8]. In most instances these movements involved individuals transiting along continuous continental coastlines rather than across island chains [8], [13]. However, a recent study using satellite telemetry off eastern Australia found that one manta ray traveled 2,441 km in 118 days and moved 155 km offshore [14]. While physical barriers, including open expanses of sea in some island nations [17] may restrict *M. alfredi,* we are now left to consider that *M. alfredi* under certain circumstances or in some locations may undertake regular longer distance movements. Understanding the regular migratory routes of *M. alfredi* in Indonesia could have important implications to national management plans for this species.

The reasons underpinning movements between aggregation sites within Indonesia are yet to be fully determined but could be linked with environmental (seasons, tidal influence, temperature, moon phase), biological (food availability, mating), or ecological (critical habitat use, habit disruption) factors. Dewar *et al.* (2008) suggest that the north to south movements within the Komodo National Park coincide with seasonal changes and monsoon shifts and the associated reduction of productivity [11]. Jaine *et al.* (2012) and Anderson *et al.* (2011) also provide evidence that manta ray visitations to Lady Elliot Island, Australia [12] and movements in the Maldives [23], respectively, are seasonally driven and linked to productivity. A comprehensive study of the feeding ecology of this species in Indonesia is therefore warranted and could assist in the understanding of their migratory behaviors in the region.

### Highlighting threats

Marine protected areas are seen as the hallmark of conservation and their benefits have been extensively reviewed in [24]–[26]. Creating comprehensive protection for migratory species can often prove challenging, with many marine species using both inshore aggregation areas and offshore habitats [14], [27]–[28]. Regional management strategies for these species therefore need to incorporate seasonal or regular migratory behavior. This study demonstrated connectivity between several aggregation areas for *M. alfredi* in Indonesia and highlighted the need to work towards more comprehensive regional protection for these species in an area that is currently fraught with anthropogenic threats.

Considering the likely trajectory of individuals moving between aggregation sites, manta rays may be crossing important shipping corridors, increasing their likelihood for boat strike. One of the documented migrations in this study required an individual to pass through the busy shipping corridor located between Mansuar and Mansfield Islands in the RA region. Manta rays transiting from Nusa Penida to the Gili Islands would likely be crossing the Lombok Strait, a heavily used shipping corridor between the islands of Bali and Lombok. Graham *et al*. [27] highlighted similar concerns for manta rays using inshore habitats off the Yucatan, with busy shipping lanes overlapping the areas being utilized by satellite tagged individuals.

More over, in 2012, Indonesia ranked as the 3rd most aggressive fishing nation for manta rays [3]. One of its most productive manta ray fishing ports (Tanjung Luar) lies directly in between NP and WM & K regions, while the other (Lamakera) lies about 380 km east of WM & K [3]. Given their migratory ability, it is reasonable to assume that *M. alfredi* using NP, the Gili Islands, and WM & K regions are at risk from targeted fishing when traveling outside of these discrete protected sanctuaries. Recent legislation has shown that Indonesia is taking the right steps forward to safeguarding their manta rays by prohibiting fishing throughout their entire exclusive economic zone (an area of over 6 million square kilometers). In reality however, it may be a long time before all manta ray fisheries in Indonesia are completely shut down. Additional regional research on the movements and habitat use of manta species will further elucidate trends in their behavior aiding to strengthen management strategies.

Comprehensive management of manta ray populations within Indonesia would be a critical step towards developing a sustainable tourism industry for these animals in the country. Annually manta ray related tourism brings in more revenue to Indonesia (>15 million US$) than manta ray fisheries (approximately US$442,000) [1]. Conceivably manta ray tourism is still a growing industry in Indonesia as manta rays are encountered in many regions throughout the country [1]. Given the economic opportunities and benefits it is recommended that a national management strategy for this species be drafted immediately to protect remaining populations within the country.

### ‘Manta Matcher’ as a collaborative tool

Data collection for this study was enabled by the participation of citizen scientists. The sampling range and the amount of available data for this study were both enhanced by combining the work of local dive operators and interested members of the public. Citizen science data is increasingly used in scientific studies [8], [12], [13], [19], [20] as it is a cost effective and relatively fast way to collect large amounts of data. The ‘Manta Matcher’ was collaboratively developed as a tool for researchers to store, organize and analyze datasets. The automated matching algorithm built into the online interface considerably reduces the time it takes to match new data to existing catalogues and increases the accuracy of the photo-ID matching process. As the first public global online database for manta rays, ‘Manta Matcher’ provides the ideal platform for regional collaborations by consolidating different data collection efforts and allowing for more comprehensive studies to take place. Using ‘Manta Matcher’ we were able to create regional catalogues and subsequently cross-reference these catalogues with ease as well as search for matches with any previously contributed data. The ‘Manta Matcher’ database for Indonesia has quickly risen to over 820 individuals, making it one of the largest composites of identified *M. alfredi* in the world. Given the effectiveness of a matching tool like ‘Manta Matcher’ and its ability to facilitate regional and global research efforts, it is recommended that this global program become the standard for manta ray researchers.

## Supporting Information

Table S1 **Manta Matcher ID and encounter codes for key individuals displaying long-range movements.**
(DOC)
