# Correction: Running the Gauntlet: Regional Movement Patterns of *Manta alfredi* through a Complex of Parks and Fisheries

**DOI:** 10.1371/journal.pone.0115660

**Published:** 2014-12-10

**Authors:** The PLOS ONE Staff

There are errors in the Author Contributions. The correct contributions are: Conceived and designed the experiments: ESG ADM. Analyzed the data: ESG ADM. Contributed to the writing of the manuscript: ESG ADM.

Additionally, there is an error in Table 2. The second data entry under Gili Islands for Identification # INNLP0074A should begin with a left-facing arrow, not with an asterisk. Please view the correct Table 2 here.

**Table 2:** The sighting records of individual rays migrating between Nusa Penida and Gili Islands or West Manggarai & Komodo regions.

Identification #	Nusa Penida	Gili Islands	West Manggarai & Komodo	Time between Resightings (days)	Approximate Distance (km)
INNLP0031A	2009-Aug-18⇒		⇒2013-Apr-03	1324	450
	2013-Sep-07⇐		⇐2013-Apr-03	157	450
INNLP0057A	2012-Sep-22⇒	⇒2013-Jan-31		131	80
INNLP0059A	2008-Dec-25⇒		⇒2013-Mar-24	1550	450
	2013-Jun-03⇐		⇐2013-Mar-24	72	450
	2013-Dec-07⇒		⇒2014-Jan-09	33	450
INNLP0074A	2012-Mar-06⇒	⇒2013-Feb-12		343	80
	2013-Mar-22⇐	⇐2013-Feb-12		38	80
INNLP0229A	2012-Sep-13⇒		⇒2013-Jun-10	270	435
	2014-Jul-18⇐		⇐2014-Jun-04	44	435

Arrows highlight direction of movements. ⇒ west to east movements; ⇐ east to west movements.

## References

[1] Germanov ES, Marshall AD (2014) Running the Gauntlet: Regional Movement Patterns of *Manta alfredi* through a Complex of Parks and Fisheries. PLoS ONE 9(10): e110071 doi:10.1371/journal.pone.0110071 25337865 10.1371/journal.pone.0110071 PMC4206290

