# Supplementary material for: Running the Gauntlet: Regional Movement Patterns of Manta alfredi through a Complex of Parks and Fisheries
Source: PLoS One. 2014 Oct 22;9(10):e110071. doi: 10.1371/journal.pone.0110071 (PMC4206290; doi:10.1371/journal.pone.0110071)
Supplement: Table S1 — Manta Matcher ID and encounter codes for key individuals displaying long-range movements. (DOC) [file pone.0110071.s001.doc]

**Table S1. Manta Matcher ID and encounter codes for key individuals displaying long-range movements.**

| ID code | Date | Region | Encounter Code |
| --- | --- | --- | --- |
| INNLP0031A | 18-Aug-2009 | NP | cfaf2680-4e89-49b6-abf3-4f25c98383be |
|  | 2013-Apr-03 | WM&K | 140225053640269 |
|  | 2013-Sep-07 | NP | 140215100414410 |
| INNLP0057A | 2012-Sep-22 | NP | 140215102935602 |
|  | 2013-Jan-31 | GI | 140215103717312 |
| INNLP0059A | 2008-Dec-25 | NP | 140216045746838 |
|  | 2013-Mar-24 | WM&K | 140225050750022 |
|  | 2013-Jun-03 | NP | 140803160400755 |
|  | 2013-Dec-07 | NP | 140216044521164 |
|  | 2014-Jan-09 | WM&K | 140220092605656 |
| INNLP0074A | 2012-Mar-06 | NP | 140303063232127 |
|  | 2013-Feb-12 | GI | 140303064820749 |
|  | 2013-Mar-22 | NP | e3ec1e35-2be0-430f-b3c8-95d322c62781 |
| INNLP0229A | 2012-Sep-13 | NP | 140216050757852 |
|  | 2013-Jun-10 | WM&K | 140324143456906 |
|  | 2014-Jun-04 | WM&K | 140810144737224 |
|  | 2014-Jul-18 | NP | 140730051352255 |
| INRA0050A | 2014-Jan-18 | RA | 140307031118207 |
|  | 2014-Jan-21 | RA | 3045c1c0-2d82-4dfd-9b9b-c70dd56d81c6 |
|  | 2014-Jan-26 | RA | 647fe44b-2911-4754-8655-f3afa3b6c3e1 |

Region key: Nusa Penida (NP); Gili Islands (GI); West Manggarai & Komodo (WM&K)
